# Successful placement of a chest wall venous infusion port via persistent left superior vena cava: A case report

**DOI:** 10.1097/MD.0000000000039978

**Published:** 2024-10-04

**Authors:** Ziqiang Wang, Rongguo Li, Qiwei Du, Weijun Zhang, Zhenyu Wang

**Affiliations:** a Breast Surgery Department, The First People’s Hospital of Xiaoshan District, Xiaoshan Affiliated Hospital of Wenzhou Medical University, Hangzhou, China.

**Keywords:** breast cancer, persistent left superior vena cava, venous infusion port

## Abstract

**Rationale:**

Persistent left superior vena cava (PLSVC) is a rare congenital venous anomaly occurring in approximately 0.3% to 0.5% of the population. The presence of PLSVC complicates central venous catheter placement, increasing procedural risks. This case report describes the successful placement of a chest wall venous infusion port in a patient with PLSVC, offering valuable insights for managing similar cases and ensuring safer clinical outcomes.

**Patient concerns:**

A 51-year-old female, 3 weeks post-right breast cancer surgery, was admitted for her first adjuvant hemotherapy session. She requested the placement of a venous infusion port due to the prolonged duration of chemotherapy.

**Diagnoses:**

Imaging studies suggested the presence of PLSVC. Echocardiography revealed a dilated coronary sinus, and subsequent chest computed tomography and angiography confirmed the presence of PLSVC.

**Interventions:**

A chest wall venous infusion port was inserted via the left internal jugular vein. Due to the PLSVC, the catheter was adjusted to ensure proper placement.

**Outcomes:**

The patient successfully completed chemotherapy without any complications or discomfort associated with the venous port. Imaging studies, including chest X-ray and computed tomography, confirmed proper port function and catheter positioning, with no evidence of thrombosis, infection, or other related issues. The patient remained in good overall condition throughout the treatment.

**Lessons:**

Detailed preoperative evaluations, intraoperative imaging guidance, and postoperative follow-ups are crucial for the safe and effective management of PLSVC patients undergoing central venous catheter placement.

## 1. Introduction

Persistent left superior vena cava (PLSVC) is a rare congenital vascular anomaly, with an incidence of approximately 0.3% to 0.5%.^[1]^ Most patients with PLSVC are asymptomatic and are often incidentally discovered during procedures such as central venous catheterization or pacemaker implantation. The presence of PLSVC can pose challenges and increase the risk of complications during these procedures.

Anatomically, PLSVC is commonly associated with the presence of a right superior vena cava, with about 65% of cases lacking a bridging innominate vein.^[2]^ PLSVC typically drains into the right atrium (RA) via the coronary sinus (CS), but variations exist where it may drain into the left atrium, leading to right-to-left shunts and related complications. Understanding and accurately identifying PLSVC is crucial for ensuring the safety and effectiveness of such procedures.

This case report describes the successful placement of a chest wall venous infusion port via PLSVC, highlighting the special techniques and adjustments required to manage this vascular anomaly. Through a detailed account of this case, we aim to provide valuable experience and insights for clinicians, enhancing the management of similar cases in clinical practice.

## 2. Case presentation

The patient, a 51-year-old female, was admitted for her first adjuvant chemotherapy session 3 weeks after right breast cancer surgery. She had a 5-year history of hypertension. Physical examination revealed the absence of the right breast, with a well-healed 15 cm surgical scar on the right chest wall. The left breast was unremarkable, and no palpable masses or nodules were found on either chest wall. There was no significant subcutaneous fluid accumulation on either chest wall, and no enlarged lymph nodes were palpable in the bilateral axillae and subclavian regions. The admission diagnosis was invasive ductal carcinoma of the right breast (pT1N3M0). The patient was scheduled for 8 cycles of chemotherapy and requested the placement of a venous infusion port due to the prolonged duration of chemotherapy. Given the previous right breast surgery, we opted for the insertion of the infusion port via the left internal jugular vein.

## 3. Surgical procedure and postoperative condition

The patient was placed in a supine position with her head turned to the right. Following routine disinfection and draping, 1% lidocaine was used for local infiltration anesthesia. A 0.5 cm incision was made at the posterior edge of the left sternocleidomastoid muscle. Blunt dissection was performed with hemostatic forceps to expose the subcutaneous tissue and platysma muscle. Under ultrasound guidance, the left internal jugular vein was punctured successfully with a puncture needle, and a guidewire was inserted, followed by a sheath introducer. At the anterior chest wall, 2 cm below the clavicle, a 2 cm long incision was made. The skin and subcutaneous tissue were dissected to create a subcutaneous pocket measuring approximately 3.0 × 2.0 cm between the pectoralis major fascia and the subcutaneous fat. The port base was placed in the subcutaneous pocket. A tunneling needle was used to create a subcutaneous tunnel from the port site to the puncture site, and the catheter was guided through the tunnel. The catheter length was measured and trimmed to 27 cm, including 18 cm from the left side and 9 cm from the tunneling needle. The excess catheter was trimmed, and the catheter was connected to the port base. The incision was closed with absorbable sutures.

**Figure 1.**
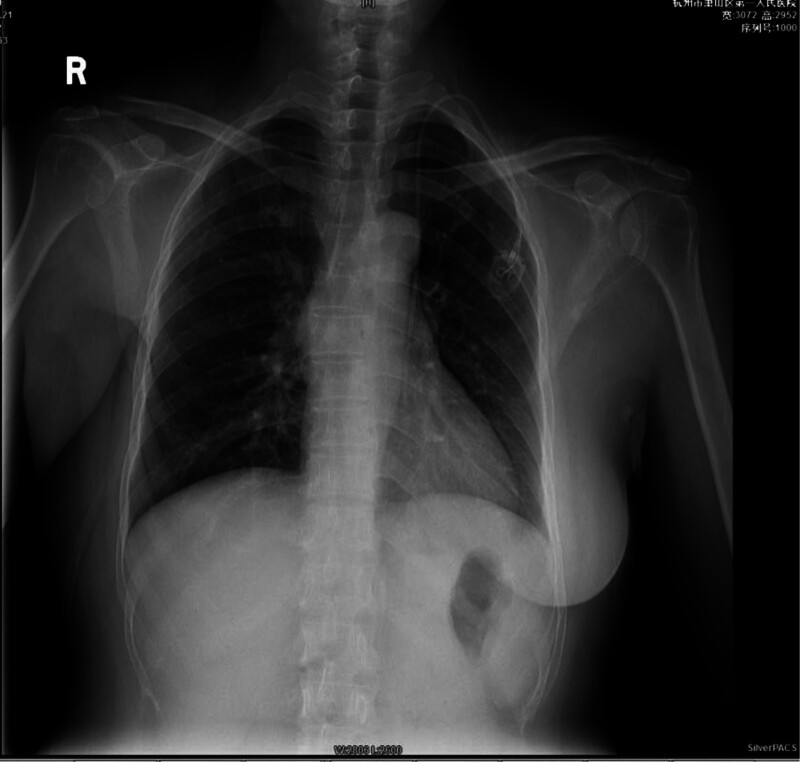
The shadow of the left thoracic catheter is located in the 8th thoracic vertebra.

**Figure 2.**
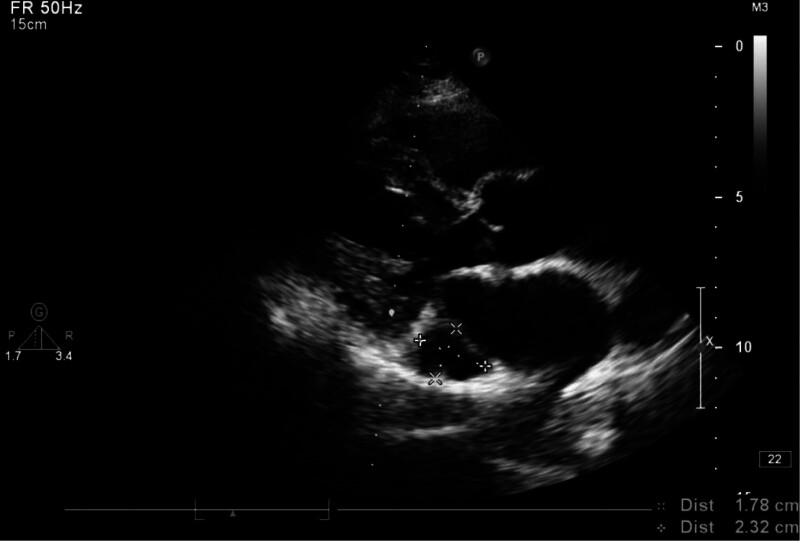
Echocardiogram shows coronary sinus dilatation.

**Figure 3.**
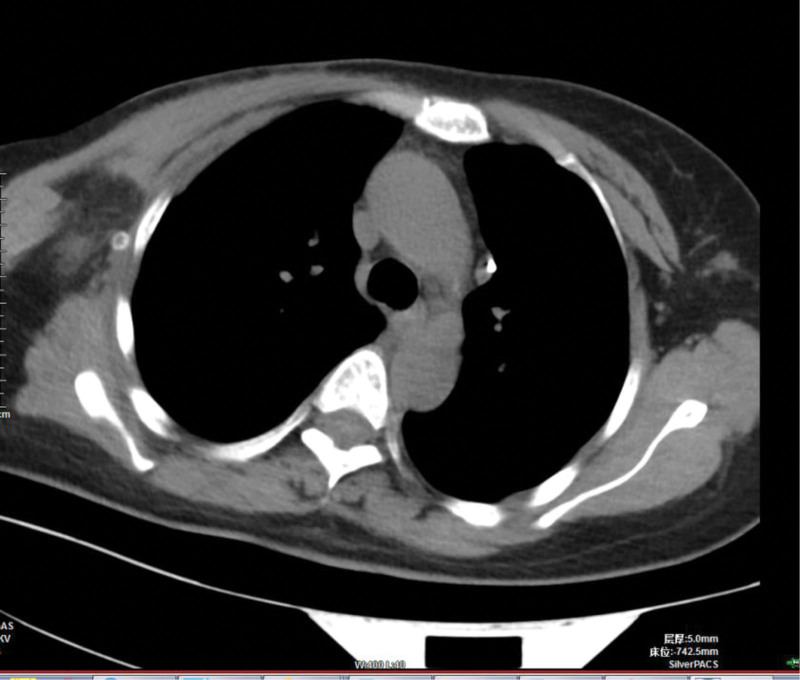
CT: persistent left superior vena cava. CT = computed tomography.

**Figure 4.**
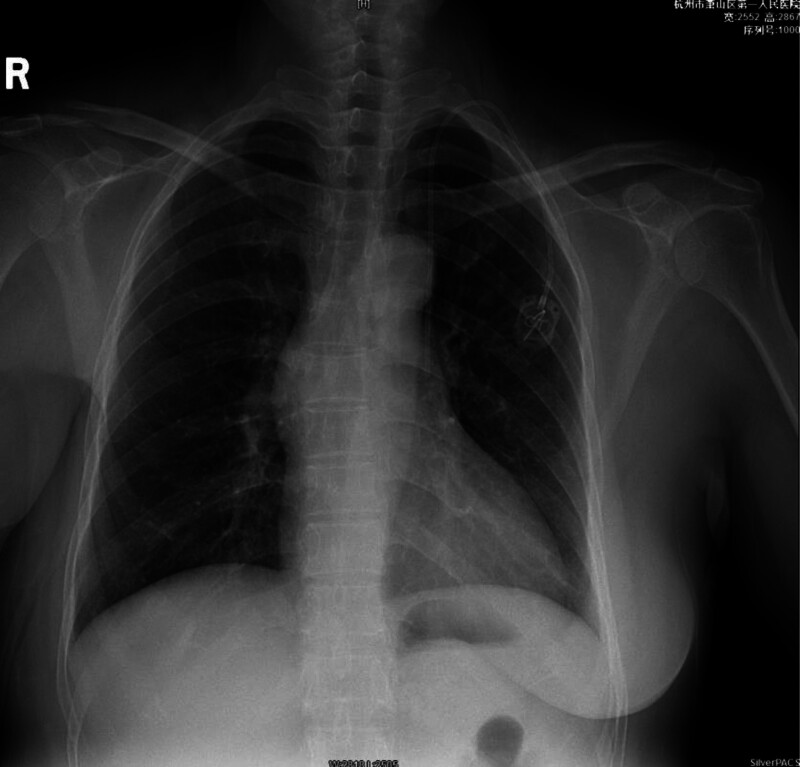
Catheter shadow in the left chest, with the end of the catheter approximately at the level of the 7th thoracic vertebra.

Postoperative chest X-ray showed the catheter tip in the left chest at the level of the eighth thoracic vertebra, suggesting the presence of a PLSVC (Fig. 1). An immediate echocardiogram revealed a dilated CS with a diameter of approximately 2 cm, and the catheter tip was located near the CS (Fig. 2). A subsequent chest computed tomography (CT) confirmed the PLSVC with the catheter in place (Fig. 3). After an interdisciplinary discussion, it was concluded that the patient had a dual superior vena cava with a PLSVC, a vascular anomaly. The catheter was correctly placed as per normal vascular anatomy, but the tip was located near the CS. A decision was made to adjust the left chest wall infusion port. Local anesthesia with 1% lidocaine was administered, the port was repositioned, and the catheter length was adjusted by trimming 4 cm. A repeat chest X-ray confirmed the catheter tip at the level of the seventh thoracic vertebra (Fig. 4).

**Figure 5.**
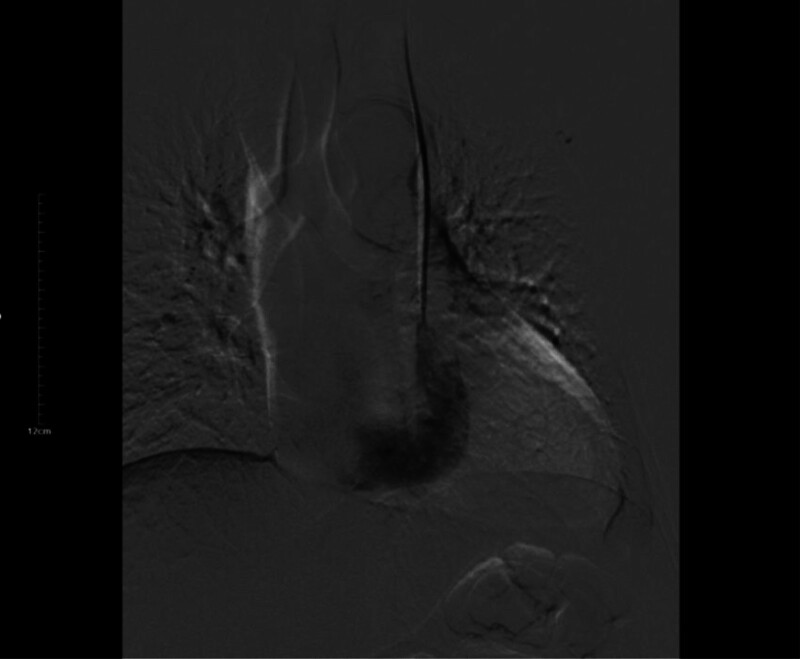
Angiography shows that contrast agent is directly introduced into the right atrium through the catheter.

**Figure 6.**
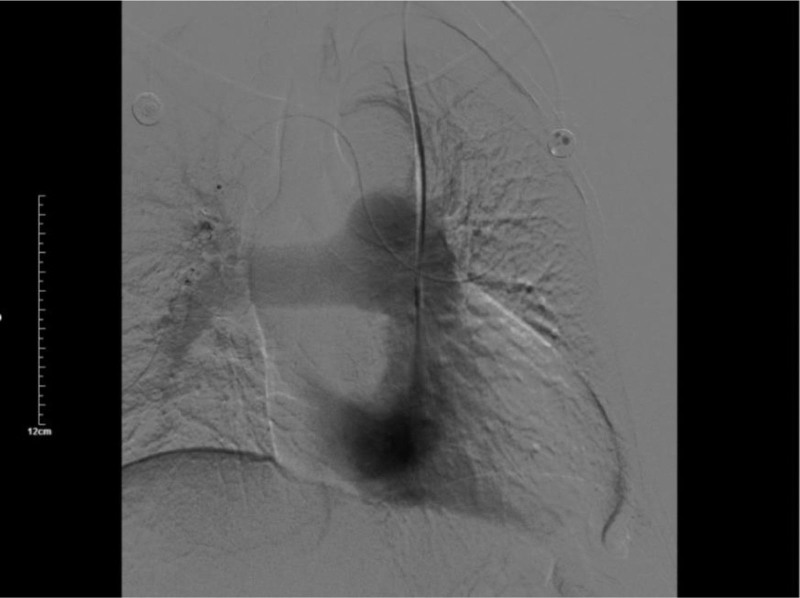
Pulmonary artery enhancement was seen in the delayed phase, but no premature enhancement was seen in the left heart.

The next day, an iodinated contrast agent was used for left superior vena cava angiography. Digital subtraction angiography fluoroscopy showed the left chest wall infusion port in place, with the catheter running along the left side of the thoracic aorta and the tip positioned at approximately the level of the seventh thoracic vertebra. The infusion port was connected to a high-pressure injector, and the contrast agent was injected through the catheter, directly entering the RA. Delayed phase imaging showed visualization of the pulmonary artery without premature visualization of the left heart. The angiography was successful, confirming the catheter’s good position, directly entering the RA, and further verifying the presence of a PLSVC (Figs. 5 and 6). The catheter tip was located in the middle to lower third of the left superior vena cava, with stable hemodynamics, allowing continued use of the chest wall infusion port. The patient completed the first chemotherapy session with no significant discomfort and was given rivaroxaban tablets, 1 tablet orally daily, for anticoagulation treatment.

## 4. Discussion

PLSVC is a relatively rare congenital venous anomaly, with an incidence of approximately 0.3% to 0.5%.^[1]^ Most patients with PLSVC (up to 90%) have bilateral superior vena cavae. Among these patients with “dual” superior vena cavae, 65% lack a bridging innominate vein, and the right superior vena cava is usually smaller.^[2]^ PLSVC is typically asymptomatic and often discovered incidentally during procedures like central venous catheterization or pacemaker implantation,^[3]^ posing unique challenges and risks. The discovery of PLSVC during the implantation of a left chest wall infusion port in this case represents the first reported instance in Zhejiang Province and is among the few documented cases in China, highlighting its rarity.

Based on hemodynamic characteristics, PLSVC can be classified into 4 types. Type I PLSVC drains into the RA via the CS, accounting for about 90% of all PLSVC cases. These patients usually do not have other cardiovascular anomalies, allowing continued use of the catheter when the tip is correctly positioned. Type II PLSVC also drains into the RA via the CS but has an additional open connection to the left atrium, causing right-to-left shunt. Type III PLSVC drains directly into the left atrium, causing right-to-left shunt. Type IV PLSVC presents as CS atresia, where the CS is obstructed or absent, leading venous blood to enter the left atrium and mix with arterial blood, often resulting in varying degrees of cyanosis.^[4]^ During catheter placement, even minimal air entering the catheter can cause fatal air embolism in patients with type II, III, and IV PLSVC, necessitating catheter removal and referral to cardiothoracic surgery. This case is type I PLSVC, where the patient’s PLSVC drains into the RA via the CS without other cardiovascular anomalies, allowing safe catheter use.

In most PLSVC patients, the right and left superior vena cavae coexist, accounting for 80% to 90% of PLSVC cases. These anatomical variations are often identified by imaging examinations showing CS enlargement. Detecting an enlarged CS during cardiac ultrasound usually indicates the presence of PLSVC.^[5]^ This finding is clinically significant as it not only confirms PLSVC but also provides clues to other potential cardiovascular abnormalities. In this case, the patient’s cardiac ultrasound showed a significantly enlarged CS measuring approximately 1.78 × 2.32 cm, further confirming PLSVC. This imaging evidence is crucial for accurate diagnosis and management of PLSVC, aiding in developing an appropriate treatment plan to ensure patient safety and efficacy. Combining these imaging findings allows a better understanding of the patient’s specific anatomical structure, ensuring appropriate measures during treatment to reduce complications.

When placing a central venous catheter, the ratio of the catheter diameter to the vein diameter significantly impacts the safety of catheter placement. To reduce the risk of thrombosis, the literature recommends maintaining the ratio of the catheter diameter to the vein diameter below 45%.^[6]^ An oversized catheter can obstruct blood flow and increase thrombosis risk. In this case, the patient’s PLSVC had a diameter >12 mm, while the infusion port catheter had a diameter of 2.21 mm, resulting in a ratio well below 45%, ensuring safe catheter placement. Based on ultrasound examination results and related literature recommendations, this ratio ensures smooth blood flow after catheter placement, reducing the risk of complications. Therefore, the catheter placement process in this case was successful and safe, allowing the patient to proceed smoothly with subsequent chemotherapy treatment.

Ensuring accurate positioning of the catheter tip is crucial for avoiding complications and ensuring optimal function during central venous catheter placement. PLSVC is a relatively rare vascular anomaly that can complicate the catheter placement process. When PLSVC is identified, the recommended approach is to position the catheter tip in the middle to lower third of the PLSVC.^[4]^ Intraoperative imaging (such as ultrasound or X-ray) is essential to confirm the catheter position. Once the catheter approaches the RA, it should be gently retracted by approximately 3 cm to ensure that the catheter tip is properly anchored in the middle to lower third of the left superior vena cava. This method minimizes the risk of complications and ensures smooth administration of chemotherapy or other intravenous treatments. Proper positioning is vital to avoid potential issues such as thrombosis, infection, and inadequate drug delivery. Therefore, operators must be familiar with the anatomical variations related to PLSVC and apply guiding and observation techniques during catheter placement to ensure patient safety and successful catheter placement.

For PLSVC patients, detailed preoperative evaluations, including enhanced CT, cardiac ultrasound, or magnetic resonance imaging, are essential to identify anatomical variations. Intraoperative real-time imaging guidance (such as fluoroscopy or ultrasound) should be used to ensure correct catheter positioning, avoid errors, and reduce complications. Postoperative imaging examinations should be conducted to confirm catheter position, and regular follow-ups are necessary to assess hemodynamics and prevent complications such as thrombosis.^[7]^ In this case, the patient required long-term chemotherapy after right breast cancer surgery, with no abnormal venous anatomy detected preoperatively. Postoperative digital radiography examination revealed the catheter position in the left chest at the level of the seventh thoracic vertebra (comparable to normal right-sided catheter placement). Further cardiac ultrasound and chest CT confirmed PLSVC. Left superior vena cava angiography showed the contrast agent directly entering the RA through the catheter, with delayed visualization of the pulmonary artery and no premature visualization of the left heart. This confirmed PLSVC with stable hemodynamics.

Catheter placement in PLSVC may lead to complications such as arrhythmias, venous stenosis, and thrombosis. Although cardiac arrhythmias, including atrial and ventricular fibrillation, have been reported in patients with PLSVC, these arrhythmias are typically caused by the dilatation of the CS, which may stretch the atrioventricular node and bundle of His, especially in cases where the right superior vena cava is absent. However, in our case, no such arrhythmias or related complications were observed postoperatively.^[8]^ To ensure safety, the left persistent internal jugular vein should be evaluated for its connection to the RA via the CS and its hemodynamic status. The catheter tip should be adjusted to the middle to lower third of the left superior vena cava to avoid potential complications. After these detailed examinations and adjustments, the patient was given prophylactic anticoagulation therapy with rivaroxaban tablets, 1 tablet daily, and successfully completed chemotherapy without significant discomfort. These steps highlight the complexity and necessary precautions for central venous catheter placement in PLSVC patients to ensure safe and effective treatment. Regular follow-up and imaging assessments are crucial for early detection and management of potential complications.^[9]^

The presence of PLSVC can pose challenges for central venous access placement. Detailed preoperative evaluation and intraoperative imaging guidance are key to ensuring safe catheter placement. With appropriate management strategies, complications associated with PLSVC can be effectively reduced, improving surgical success rates, and patient quality of life.^[10]^

## 5. Limitations

The main limitation of this study is that it is a single case report of a rare condition, which limits the generalizability of the findings. Although this case demonstrates the successful placement of a venous infusion port in a patient with PLSVC, further studies involving larger cohorts and longer follow-up periods are needed to fully assess the long-term safety and efficacy of this technique in similar cases.

## 6. Conclusions

PLSVC is a rare but significant anatomical variation that can complicate central venous access procedures. This case report demonstrates that with detailed preoperative planning, intraoperative imaging guidance, and careful postoperative management, it is possible to safely and effectively place a chest wall venous infusion port in patients with PLSVC. Recognizing the presence of PLSVC and understanding its implications are crucial for preventing complications and ensuring successful outcomes. This case contributes to the growing body of literature on managing PLSVC and provides valuable insights for clinicians facing similar challenges.

## Acknowledgments

The authors would like to credit the patient for her participation in this case study.

## Author contributions

**Writing—original draft:** Ziqiang Wang.

**Writing—review & editing:** Ziqiang Wang.

**Data curation:** Rongguo Li, Qiwei Du, Weijun Zhang, Zhenyu Wang.

**Investigation:** Rongguo Li.

**Conceptualization:** Zhenyu Wang.
